# Risk factors for depression in asthmatic individuals: Findings from NHANES (2005–2018)

**DOI:** 10.1371/journal.pone.0287336

**Published:** 2023-06-15

**Authors:** Huan Yang, Ping Lin, Zongan Liang

**Affiliations:** Department of Respiratory and Critical Care Medicine, West China School of Medicine and West China Hospital, Sichuan University, Chengdu, China; Tower Health - Phoenixville Hospital, UNITED STATES

## Abstract

**Background:**

The risk factors for depression in asthma are still unclear. The objective of this study was to identify the risk factors associated with depression in asthmatic individuals.

**Methods:**

We used data from the 2005–2018 National Health and Nutrition Examination Survey (NHANES). Univariate analysis and multivariate logistic regression analyses were used to identify risk factors for depression and calculate unadjusted and adjusted odds ratios (ORs) and 95% confidence intervals (CIs).

**Results:**

A total of 5,379 asthmatic participants were included. Of these subjects, 767 individuals had depression, and 4,612 individuals had no depression. Univariate analysis and multivariate analyses suggested that asthmatic individuals with smoking (OR 1.98, 95% CI 1.19–3.29), hypertension (OR 2.73, 95% CI 1.48–5.04), and arthritis (OR 2.83, 95% CI 1.53–5.22) were more likely to have depression. Asthmatic individuals who had more than a high school education had lower depression risk than those with less than a high school education (OR 0.55, 95% CI 0.30–0.99). Increasing age was also associated with decreased depression risk (OR 0.97, 95% CI 0.95–0.99).

**Conclusions:**

Depression was more likely in asthmatic individuals with smoking, hypertension, and arthritis and less likely in individuals with higher education and increasing age. These findings could improve the identification of target populations for effective interventions to improve the mental health of asthmatic individuals.

## Introduction

Asthma is a complex chronic inflammatory disorder of the conducting airways characterized by bronchial hyper-responsiveness and variable airflow obstruction [1]. It affects about 20 million individuals in the United States and over 300 million individuals worldwide and accounts for 1 in every 250 deaths in the world [2]. The cost of asthma management is $USD 1,900 in Europe and $USD 3,100 in the United States per person per year, indicating that asthma is associated with a significant economic and social burden [3].

Asthmatic individuals frequently had comorbidities, such as psychiatric symptoms and mental disorders [4]. Epidemiological studies showed that about 5% of adults worldwide suffer from depression [5], but this value for asthmatic individuals was more than 10% [6, 7]. Asthmatic individuals with depression were associated with a decreased level of asthma control and poor quality of life compared to those without depression [8, 9]. Treating depression had been shown to improve asthma control, corticosteroid use, and asthma exacerbations [10]. Therefore, it is necessary to identify and treat depression in asthmatic individuals.

Knowing the risk factors related to depression in asthmatic patients will help physicians recognize and intervene early in depression. However, to our best knowledge, there are no studies that have explored the risk factors for depression in asthmatic individuals. The risk factors of depression in asthma are still unclear. The objective of this study was to identify the risk factors (including modifiable factors) associated with depression in asthmatic individuals using a nationally representative sample from the National Health and Nutrition Examination Survey (NHANES).

## Methods

### Study population

The NHANES, conducted by the National Center for Health Statistics (NCHS) of the Centers for Disease Control and Prevention (CDC), was a nationally representative survey assessing the health and nutritional status of the U.S. civilian population [11]. NHANES data was based on household interviews and standardized physical examinations of survey participants. Each participant was weighted based on the portion of the US population that the individual represents. The protocol of NHANES was approved by the NCHS Institutional Review Board, and written informed consent was obtained from all participants.

For this study, we included data from NHANES participants from 2005 to 2018. Participants were excluded from analyses if they were without asthma or with missing information on the diagnosis of depression. Asthma relied on self-reporting of a doctor’s diagnosis. Participants were asked the questions: “Has a doctor ever told you that you have asthma?” together with “Do you still have asthma?” in interviews. In this study, asthma was defined by an affirmative answer to both questions [12].

### Covariates’ assessment

Information on age, sex, race, weight, height, education, smoking status, and drinking status was obtained by self-reported. Participants were asked the question “Do you now smoke cigarettes”. Participants who selected either “every day” or “some days” were defined as smokers. Participants who reported “not at all” were defined as nonsmokers. Drinking was defined by using the question “Had at least 12 alcohol drinks/lifetime?”. Participants who reported “yes” were defined as drinkers. Participants who responded with “no” were defined as nondrinkers. Comorbidities relied on self-reporting of a doctor’s diagnosis. Hypertension, diabetes mellitus, coronary heart disease, congestive heart failure, stroke, arthritis, emphysema, chronic bronchitis, and cancer were included as covariates in that they had been found to be associated with depression.

### Depression assessment

In NHANES 2005–2018, the Patient Health Questionnaire-9 (PHQ-9), a nine-item depression screening instrument, was used to measure depression symptoms over the past 2 weeks. The questions were asked at the Mobile Examination Center by trained interviewers. Each instrument was given a point ranging from 0 to 3 based on the response categories "not at all," "several days," "more than half the days," and "nearly every day.” Total PHQ-9 score ranges from 0 to 27 and ≥ 10 was regarded as depression [13]. Data from participants ≥18 years were included in the online data file (Depression Screener (DPQ_J)), so all eligible participants in this study were 18 years or older.

### Statistical methods

The Baseline clinical characteristics of participants were described according to depression status. Continuous variables were described using weighted means and standard deviations and analyzed using weighted linear regression models. Categorical variables were described using weighted percentages and analyzed using weighted chi-square tests. Univariate analysis and multivariate logistic regression analysis were used to identify risk factors for depression and calculate unadjusted and adjusted odds ratios (ORs) and 95% confidence intervals (CIs). Age, gender, race, body mass index (BMI), education, cigarette smoking, drinking, hypertension, diabetes mellitus, coronary heart disease, congestive heart failure, stroke, arthritis, emphysema, chronic bronchitis, and cancer were included in both univariate analysis and multivariate logistic regression. All statistical analyses were done using EmpowerStats statistical software 2.0 (http://www.empowerstats.com, X&Y Solutions, Inc., Boston, MA) and Stata MP 14.0 with appropriate sampling weights. A two-tailed P value <0.05 was considered statistically significant.

## Results

### Participant characteristics

During the 2005~2018 NHANES cycles, 70,190 participants were interviewed. We excluded participants without asthma (60,211) and with missing information on the diagnosis of depression (4,600). Finally, a total of 5,379 asthmatic participants were included. Of these subjects, 767 individuals had depression, and 4,612 individuals had no depression. There are significant differences between subjects with and without depression (Table 1).

**Table 1:** Patient baseline demographic and clinical characteristics.

Characteristic	Without depression (N = 4612)	With depression (N = 767)	P value
Age(y)	43.9 ± 17.7	45.5 ± 15.7	0.0269
Man (%)	43.0	33.2	<0.001
BMI (kg/m2)	29.9 ± 7.8	32.0 ± 9.1	<0.001
Race (%)			0.0104
Mexican American	5.4	4.7	
Other Hispanic	4.9	8.1	
Non-Hispanic White	69.6	65.7	
Non-Hispanic Black	13.0	14.1	
Other races	7.1	7.4	
Education (%)			<0.001
Less than high school	12.6	27.3	
High school or GED	22.0	25.7	
More than high school	65.3	47.0	
Smoking (%)	45.1	65.6	<0.001
Drinking (%)	67.1	64.8	<0.001
Hypertension (%)	33.2	50.8	<0.001
Diabetes mellitus (%)	10.0	20.5	<0.001
CHD (%)	3.7	9.3	<0.001
CHF (%)	3.5	8.9	<0.001
Stroke (%)	3.5	10.5	<0.001
Arthritis (%)	31.6	55.7	<0.001
Emphysema (%)	4.3	12.4	<0.001
Chronic bronchitis (%)	17.4	32.7	<0.001
Cancer (%)	11.4	14.3	0.0291

Abbreviations: GED, general educational development; CHD, coronary heart disease; CHF, congestive heart failure.

### Depression risk

In univariable logistic analyses (Table 2), the likelihood of depression was highest in females, Other Hispanic, and individuals with increasing age, obesity, less than high school education, current smoking, hypertension, diabetes mellitus, coronary heart disease, congestive heart failure, stroke, arthritis, emphysema, and chronic bronchitis. Further multivariable analyses confirmed that asthmatic individuals with smoking (OR 1.98, 95% CI 1.19–3.29), hypertension (OR 2.73, 95% CI 1.48–5.04), and arthritis (OR 2.83, 95% CI 1.53–5.22) were more likely to have depression (Table 3). Asthmatic individuals who have more than a high school education had lower depression risk than those with less than a high school education (OR 0.55, 95% CI 0.30–0.99). Increasing age was also associated with decreased depression risk in asthmatic individuals (OR 0.97, 95% CI 0.95–0.99).

**Table 2:** Univariate logistics analysis showing odds ratios (ORs) for variables affecting risk of depression.

Characteristics	OR	95% CI	p Value
Age	1.01	1.00 to 1.01	0.045
Gender (female)	1.52	1.23 to 1.88	<0.001
Race			
Non-Hispanic White	1[Reference]		
Mexican American	0.93	0.66 to 1.30	0.663
Other Hispanic	1.73	1.29 to 2.31	<0.001
Non-Hispanic Black	1.15	0.92 to 1.43	0.223
Other races	1.11	0.78 to 1.58	0.560
Weight			
Normal weight	1[Reference]		
Underweight	1.67	0.52 to 5.37	0.392
Overweight	0.99	0.73 to 1.36	0.977
Obesity	1.61	1.24 to 2.09	<0.001
Education			
Less than high school	1[Reference]		
High school or GED	0.54	0.41 to 0.70	<0.001
More than high school	0.33	0.26 to 0.42	<0.001
Current smoking	2.32	1.78 to 3.02	<0.001
Drinking	0.90	0.62 to 1.31	0.588
Hypertension	2.07	1.69 to 2.54	<0.001
Diabetes mellitus	2.32	1.82 to 2.97	<0.001
CHD	2.70	1.77 to 4.13	<0.001
CHF	2.69	1.85 to 3.91	<0.001
Stroke	3.25	2.27 to 4.63	<0.001
Arthritis	2.72	2.20 to 3.36	<0.001
Emphysema	3.13	2.26 to 4.32	<0.001
Chronic bronchitis	2.31	1.85 to 2.89	<0.001
Cancer	1.30	0.96 to 1.77	0.091

Abbreviations: GED, general educational development; CHD, coronary heart disease; CHF, congestive heart failure.

**Table 3:** Multivariate logistics analysis showing odds ratios (ORs) for variables affecting risk of depression.

Characteristics	OR	95% CI	p Value
Age	0.97	0.95 to 0.99	0.010
Gender (female)	1.18	0.67 to 2.09	0.573
Race			
Non-Hispanic White	1[Reference]		
Mexican American	1.24	0.43 to 3.63	0.690
Other Hispanic	1.50	0.65 to 3.47	0.345
Non-Hispanic Black	0.82	0.45 to 1.51	0.527
Other races	1.41	0.53 to 3.74	0.489
Weight			
Normal weight	1[Reference]		
Underweight	0.69	0.17 to 2.81	0.600
Overweight	1.33	0.60 to 2.94	0.480
Obesity	0.99	0.50 to 1.97	0.989
Education			
Less than high school	1[Reference]		
High school or GED	0.62	0.33 to 1.19	0.150
More than high school	0.55	0.30 to 0.99	0.045
Current smoking	1.98	1.19 to 3.29	0.008
Drinking	1.06	0.59 to 1.87	0.854
Hypertension	2.73	1.48 to 5.04	0.001
Diabetes mellitus	1.03	0.50 to 2.13	0.937
CHD	0.92	0.34 to 2.44	0.868
CHF	1.04	0.41 to 2.50	0.941
Stroke	1.81	0.80 to 4.09	0.157
Arthritis	2.83	1.53 to 5.22	0.001
Emphysema	0.62	0.28 to 1.42	0.262
Chronic bronchitis	1.52	0.84 to 2.74	0.165
Cancer	0.72	0.37 to 1.40	0.338

Abbreviations: GED, general educational development; CHD, coronary heart disease; CHF, congestive heart failure.

## Discussion

This study investigated the risk factors for depression in asthmatic individuals in a nationally representative sample of the U.S. population. The results showed that depression was more likely in asthmatic individuals with smoking, hypertension, and arthritis. Asthmatic individuals with higher education and increasing age were less likely to have depression.

Cigarette smoking remained one of the most important public health problems in the world, with heavy social, functional, and economic burdens [14, 15]. There was increasing evidence to suggest that smoking was associated with an increased risk of mental illnesses such as depression and anxiety [16]. Structural brain changes, inflammation and immune activation, increased oxidative and nitrosative stress, mitochondrial dysfunction, neurotransmitter systems and epigenetic alterations in smokers might have a role in the development of depression [17]. Asthma itself might contribute to a greater risk for depression and smoking would further increase the risk of depression in asthmatic individuals [18]. Asthmatic individuals should be earnestly advised not to start smoking [19]. McClave AK, et al. revealed that smoking cessation might alleviate depressive symptoms and reduce the incidence of depression [20], but a lot of studies found that quitting smoking would lead to increased depression and anxiety due to the withdrawal of nicotine [21]. Therefore, smoking cessation among asthmatic smokers should be under the guidance of doctors to avoid the withdrawal of nicotine.

In the present study, the incidence of depression was 14.2% in total asthmatic participants, whereas the incidence of depression increased to 19.9% in participants with asthma and hypertension. There was increasing evidence suggesting that there was a positive association between depression and hypertension. García-Fabela L, et al. found that hypertension was an independent predictor of depression among community-dwelling elders (adjusted OR 1.18; 95% CI,1.01–1.40) [22]. Meng, Lin, et al. found that depression increased the risk of hypertension (adjusted relative risk 1.42, 95% CI,1.09–1.86) [23]. Hypertension and depression had many common biological characteristics, such as systemic inflammation, metabolic syndrome, and dysregulation of the hypothalamic–pituitary–adrenal axis [24, 25]. Therefore, hypertension and depression might be risk factors for each other [23]. Asthmatic individuals with comorbid hypertension were associated with worse asthma control and better control of blood pressure might alleviate asthma symptoms [26]. Antidepressant treatment had been found to potentially improve hypertension and asthma control [27, 28]. Therefore, awareness, treatment, and control of depression might be of value in the management of asthma [29].

Comorbid depression was common with immune-mediated inflammatory diseases due to shared pathophysiological mechanisms between the brain and peripheral immune and molecular responses [30]. Raised peripheral cytokines and chemokines (inter-leukin-1β [IL-1β], IL6, C-reactive protein [CRP], tumor necrosis factor-α [TNFα], CXCR3, and CXCL10) due to rheumatoid arthritis might change the immune status and function of the central nervous system via humoral and neuronal routes of communication [31]. It was shown that depression was the most frequent comorbidity in the rheumatoid arthritis population and it would worsen joint pain and disease activity [32, 33]. It was worth encouraging healthcare providers to screen asthmatic individuals with arthritis for depression.

In our study, we further examined whether age and education could be used to identify which asthmatic individuals were at increased risk of depression. We found that higher education was associated with a decreased risk of depression. Higher education had higher resilience in regard to strain and stresses, which might protect against depression and anxiety [34]. In addition, a low education level most often had poorer employment outcomes in adulthood, hence facing a lot of mental and physical health conditions [35]. Increasing age had also been found to be associated with a decreased depression risk (OR 0.97, 95% CI 0.95–0.99). However, this result should be interpreted carefully because the OR value and 95% CI are very close to 1. There was no clear reason why older people were more likely to develop depression. One possibility was that older people accepted the inevitability of physical illness more readily than younger people, leading to a buffer against the adverse effects of physical illness on depression [36]. Lower educational levels and younger age were also considered as risk factors for asthma [37]. Therefore, both lower educational levels and younger age were not only risk factors for asthma but also risk factors for depression in asthma. These findings did suggest that screening for depression in asthmatic individuals with lower education and younger age was necessary.

The strengths of the present study included the use of a nationally representative sample from the NHANES and the exploration of numerous potential factors pertaining to depression. Our study also had several limitations. First, as ours was a cross-sectional study, we could not confirm the direction of relationships between depression, smoking, hypertension, arthritis educational level, age, and other variables. Second, other potential covariates, such as asthma control and drug use, that could be related to depression were not collected in NHANES and therefore were not considered in our analysis. Thus, our findings should be interpreted with caution in that residual confounding could not be excluded. Lastly, many variables in this study were defined by self-reported physician diagnosis. Self-reported data were less accurate than objective records and subject to recall bias, which might limit the reliability of our results. Nonetheless, to the best of my knowledge, our study is the first to explore risk factors for depression in asthma, which might assist clinicians in identifying depression in asthmatic patients.

## Conclusions

Depression was more likely in asthmatic individuals with smoking, hypertension, and arthritis, and less likely in individuals with higher education and increasing age. These findings might assist clinicians in identifying depression in asthmatic patients.

## Abbreviations

BMI: body mass index
CDC: Centers for Disease Control and Prevention
CI: confidence interval
CRP: C-reactive protein
DPQ_J: Depression Screener
IL-1β: inter-leukin-1β
NCHS: National Center for Health Statistics
NHANES: National Health and Nutrition Examination Survey
OR: odds ratio
PHQ-9: Patient Health Questionnaire-9
TNFα: tumor necrosis factor-α
